# A Computational Framework for Investigating the Mechanical Stresses on Breast Implants Under Dynamic Loading Conditions

**DOI:** 10.1007/s10439-025-03815-x

**Published:** 2025-07-25

**Authors:** Seungkwan Lee, Ju Yeon Park, Sinwoo Park, Jung-Ju Kim, Il-Seok Jang, Ju-Dong Song, Do-Nyun Kim

**Affiliations:** 1 Department of Mechanical Engineering, Seoul National University, 1 Gwanak-ro, Gwanak-gu, Seoul, 08826 Republic of Korea; 2 Osstem Implant, 3 Magokjungang 12-ro, Gangseo-gu, Seoul, 07789 Republic of Korea; 3 Interdisciplinary Program in Bioengineering, Seoul National University, 1 Gwanak-ro, Gwanak-gu, Seoul, 08826 Republic of Korea; 4 Institute of Advanced Machines and Design, Seoul National University, 1 Gwanak-ro, Gwanak-gu, Seoul, 08826 Republic of Korea; 5 Institute of Engineering Research, Seoul National University, 1 Gwanak-ro, Gwanak-gu, Seoul, 08826 Republic of Korea

**Keywords:** Finite element analysis, Breast implants, Silicone gel, Stress prediction

## Abstract

The durability and safety of silicone breast implants remain critical concerns due to risk of rupture under long-term and dynamic loading conditions. To address these challenges, this study introduces a Finite Element Analysis (FEA)-based approach to investigate the mechanical behavior of breast implant shells under simulated clinical conditions, including compressive loading and dynamic movements such as walking. The material properties of the implant components in the computational model were characterized through an optimization process integrating 3D scan data and simulation results. Two primary loading scenarios were modeled and analyzed: compressive forces from external pressure like physical manipulation or impact, and dynamic forces induced by walking, representing typical daily activities. Simulation results identified areas of high stress concentration on implant shells, corresponding to clinically observed rupture locations. Specifically, compressive loading simulations revealed high von Mises stress levels, while walking simulations demonstrated periodic stress fluctuations after the initial transient phase, highlighting fatigue-related risks in specific regions of the implant shell. Despite limitations, such as simplified material models and generic body geometries, this study provides a robust framework for analyzing implant performance under realistic conditions. These findings offer valuable insights for improving implant design and durability, paving the way for safer, patient-specific solutions.

## Introduction

Breast surgery is one of the most common surgeries performed for women worldwide, primarily for aesthetic and reconstructive reasons [1]. In breast reconstruction following breast cancer, implants account for more than 80% of total reconstructions. The demand for breast augmentation surgery is also steadily increasing due to changing social aesthetic values [2]. Breast implants are categorized into two main types based on their internal fillings: saline implants and silicone gel implants [3].

Silicone gel implants are more popular because they offer a more natural shape and texture compared to saline implants [4–6]. Additionally, improvements in safety have made silicone gel implants the dominant choice in the global market [7, 8]. Despite these advancements, clinical trials of silicone gel breast implants over more than 10 years still report a rupture rate of about 8% [5, 6, 9]. Due to the cohesive nature of the silicone cross-linked gel, ruptures occur unnoticed by patients, which is called ‘Silent rupture’ [6, 10]. This can result in the implant materials remaining in a ruptured state within the body for several years, exacerbating symptoms such as the formation of a fibrous capsule around the implant, pain, and changes in breast size [9, 11].

Implant ruptures typically begin to occur around 5 years after implantation [10, 12]. Long-term fatigue accumulation and aging are primary causes of these ruptures, which can also be triggered by instantaneous external forces. The risk of rupture increases with the length of time the implant remains in the body [10]. Clinically, rippling—wrinkles on the surface of the implant shell—can occur due to the forces exerted by the body environment and gravity. These rippled areas often experience localized stress and fatigue accumulation, which are the leading causes of implant rupture [13].

Understanding the deformation and stress on silicone implant shells under various conditions is crucial for predicting fatigue-induced ruptures [14–17]. Fatigue analysis is widely applied across various materials and industries to assess long-term performance, identify potential failure points, and optimize designs for durability and safety [18, 19]. In the context of breast implants, fatigue analysis provides insights into long-term durability, helping to identify failure-prone areas and enhance material and design to reduce rupture risks. This approach is essential for developing safer and more effective implants, ultimately minimizing health complications associated with implant ruptures.

Despite its significance, computer-aided simulations to predict the stress accumulation in areas where rippling occurs have been notably lacking. Existing studies primarily utilize 2-dimensional simulation [20] or focus on direct compression of the implant, without adequately reflecting clinical conditions [21].

In this study, as the first step of fatigue analysis in breast implants, Finite Element Analysis (FEA) is employed to predict the stress within the implant shell. It is widely used for various purposes under loading conditions, providing detailed insights into material behavior, stress distribution, and potential failure points in a wide range of applications [22–26]. For a simplified analysis, the materials used in fabricating the implants are assumed to behave elastically without viscosity. An optimization process that compares results from FEA with 3D scanning data is used to characterize the assumed material model. The geometry and loading conditions of the implant are modeled under clinical conditions specifically considering the loadings experienced during daily activities, such as compression and the stresses induced by a patient’s movements, including walking.

By setting these realistic conditions, we aim to create a more accurate simulation of the stresses and strains that the implants would endure in real-life scenarios. This approach not only helps in understanding how these implants will perform over time but also assists in identifying critical areas where material improvements or design modifications may be necessary to enhance the overall durability and safety of breast implants.

## Materials and Methods

### Geometry of Implant

**Fig 1:**
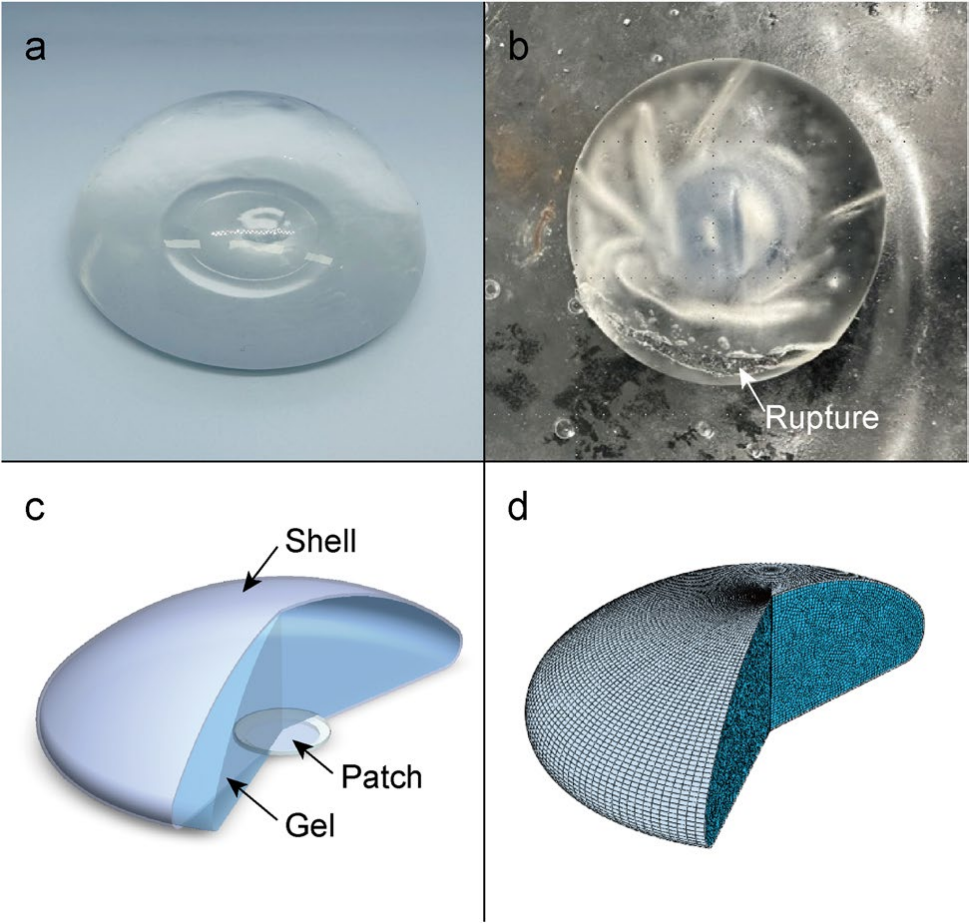
**a** Silicone breast implant showing a smooth, undamaged surface. **b** Silicone breast implant with a rupture site, highlighted by stress-induced material failure. **c** Schematic representation of the breast implant structure, showing the shell, gel, and patch regions. **d** Finite element analysis model of the breast implant, illustrating the meshed computational structure used for mechanical simulations

The geometry of breast implants consists of three main components: the shell, the gel, and the patch as shown in Fig 1c. Each component plays a distinct role in the structural and functional integrity of the implant. The shell is a thin, flexible envelope made of silicone elastomer that encloses the gel material and provides the outer shape of the implant. The gel, which fills the shell, serves to enlarge the breast volume and define the implant’s shape and softness. The patch is used to seal the opening that results during the shell manufacturing process. It ensures the formation of a completely enclosed pocket, preventing gel leakage and contributing to the implant’s safety and durability. The patch is composed of the same silicone elastomer material as the shell and is typically bonded using thermal compression. Its thickness is also equivalent to that of the shell.

In the FEA model, to simplify the simulation, the patch and shell are unified into a single shell geometry. Naturally, this simplification introduces a geometric overlap at the junction where the patch is thermally bonded to the shell during the manufacturing process. In reality, this region may exhibit a slightly different thickness compared to the rest of the shell. However, because the patch is typically located at the bottom of the implant—an area that, as shown in the Results section, experiences relatively low stress under both compressive and dynamic loading conditions—the effect of this variation is considered negligible in the context of this study. Moreover, since the shell and patch are made of the same material (silicone elastomer), this unification does not introduce material inconsistency. Therefore, combining the shell and patch into a uniform geometry allows for a more efficient simulation without significantly compromising mechanical accuracy.

Bra cup size is typically determined by the difference between the top breast circumference and the band (under bust) size, with implants primarily influencing cup size by increasing the top breast volume. In clinical practice, implant volume is closely associated with changes in cup size. Although the exact outcome can vary depending on individual anatomy, it is generally estimated that a 200 cc implant increases breast size by approximately one cup, a 400 cc implant by two cups, and an 800 cc implant by three cups.

In this study, four implant sizes were used to characterize the material properties for simulation: 200, 250, 400, and 560 cc. Each implant was modeled as an axisymmetric shape based on its undeformed geometry, with corresponding radii of 52.87, 52.97, 62.82, and 62.51 mm, and heights of 45.7, 57.4, 61.6, and 77.5 mm, respectively. A uniform shell thickness of 0.5 mm was applied to all models.

For simulations under compressive and walking loading conditions, a 125 cc implant was used as a representative case to demonstrate the applicability of the proposed computational framework. This implant, currently under development, features a non-uniform shell thickness ranging from 0.4 mm to 0.6 mm depending on location. In actual product development, shell thickness distribution and gel volume fraction are considered key design parameters. In the simulations under loading conditions, both parameters were fixed: the measured non-uniform shell thickness was applied, and the gel volume fraction was set to 1.0. This approach enabled a more controlled evaluation of stress and deformation behavior while maintaining practical relevance to early stage product design and analysis.

### Material Properties

The properties of the shell are determined following ASTM D412 testing standard, involving tensile testing on specific specimens [15, 27–29]. However, direct tensile testing of the gel component proved impractical due to its unsuitability in specimen form. To address this challenge, an alternative, indirect approach was employed to obtain the necessary properties of the gel material.

**Fig 2:**
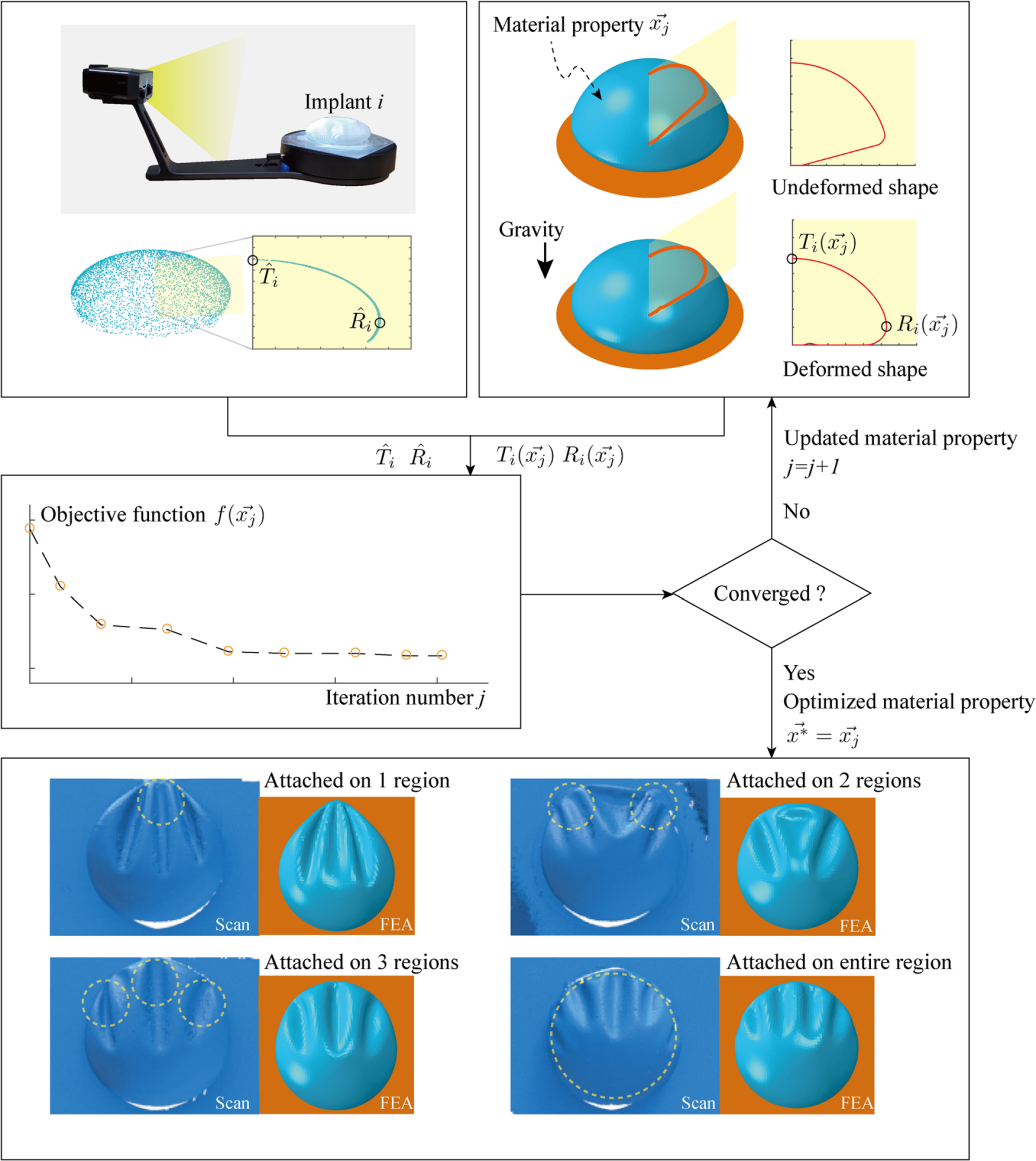
Overview of the process of characterizing material properties of breast implants through optimization. The top row illustrates the 3D scanning process to capture the geometric structure of implant *i* and computational analysis using a meshed model. These results are utilized in an optimization process where geometric fitting aligns scanned data with the computational model, minimizing discrepancies. This process iterates until the objective function *f* becomes smaller than the predefined tolerance. The characterized material properties are then validated by comparing deformation patterns between scanned implants (left) and Finite Element Analysis predictions (right) under various loading conditions, demonstrating strong agreement between experimental and simulated results

The indirect method used to determine the gel material properties and adjust the shell material properties from the tensile experiments is outlined in Fig 2. The implant is placed flat on the ground, and its external deformed shape in this state is captured using a 3D scanner (Shining 3D’s EinScan-SE model). The deformed shape of the implant in this flattened state is then simulated using FEA. Comparisons between the deformed shapes obtained from the 3D scan and FEA results were conducted and incorporated into an optimization process to characterize the material properties.

The material property $\overrightarrow{{\varvec{x}}}$, which includes the nonlinear material coefficient (**C**_**10**_) of the shell and the thermal coefficient for the gel to account for volume reduction, is used as an input in the optimization process. A neo-Hookean material model is applied to both the shell and gel materials to account for large deformations [29–31].

As previously mentioned, gel volume fraction is one of the key parameters in implant design. It is defined as the ratio between the actual gel volume filled inside the implant and the total internal volume enclosed by the shell geometry. Discrepancies between the real gel volume and the shell’s enclosed space can arise due to factors such as thermal volume shrinkage, non-ideal fulfillment during manufacturing, or incomplete sealing. A volume fraction of 1.0 represents an idealized case in which the shell is fully filled with gel and no volume reduction has occurred—an assumption commonly used for evaluating baseline mechanical performance.

When the gel volume fraction is lower, the implant is relatively under-filled, which may lead to increase its compressibility and internal movement of the shell. Under loading conditions, this can lead to surface wrinkling or folding of the shell, while frequent internal contact may induce localized stress concentrations, potentially elevating the risk of shell rupture or fatigue-related failure over time.

Conversely, a higher volume fraction (closer to 1.0) represents a more fully filled implant, offering enhanced internal support and structural stiffness. However, this can also lead to higher stress concentrations on the shell surface during deformation, particularly under compressive or dynamic loading.

Given the gel’s softness, which is significantly less stiff than the shell, the **C**_**10**_ coefficient in the neo-Hookean model for the gel is assumed to be less than 1/100 of that for the shell material. This assumption reflects the substantial difference in stiffness and ensures minimal strain energy is stored in the gel during deformation.

The objective function in the optimization process, represented by Eq. (1), integrates the discrepancies between the height and width values of the deformed implant shapes obtained from 3D scanning and those calculated using FEA simulations. The function is mathematically expressed as a weighted summation of these discrepancies, ensuring that both dimensions are appropriately considered. Specifically:

1 $$f\left( {\overrightarrow {{x_{j} }} } \right) = \sum \left( {w_{T} \left| {T_{i} \left( {\overrightarrow {{x_{j} }} } \right) - \widehat{{T_{i} }}} \right| + w_{R} \left| {R_{i} \left( {\overrightarrow {{x_{j} }} } \right) - \widehat{{R_{i} }}} \right|} \right)$$

- $f\left(\overrightarrow{{x}_{j}}\right)$: The objective function value, representing the total error between the scanned and simulated shapes.
- ${T}_{i}\left(\overrightarrow{{x}_{j}}\right)$ and $\widehat{{T}_{i}}$: Heights of the deformed implant shapes for the *i*-th implant size from FEA with material property $\overrightarrow{{x}_{j}}$ and 3D scanning, respectively.
- ${R}_{i}\left(\overrightarrow{{x}_{j}}\right)$ and $\widehat{{R}_{i}}$: Widths of the deformed implant shapes for the *i*-th implant size from FEA with material property $\overrightarrow{{x}_{j}}$ and 3D scanning, respectively.
- *w*_*T*_ and *w*_*R*_: Weight factors for the height and width discrepancies, respectively, which are used to control their relative contributions to the optimization.

At each iteration *j*, the material property vector $\overrightarrow{{{\varvec{x}}}_{{\varvec{j}}}}$ is adjusted to minimize the function $f\left(\overrightarrow{{x}_{j}}\right)$, systematically reducing the discrepancies between the scanned and simulated results. This iterative process continues until the objective function value converges within a predefined tolerance, ensuring that the final optimized material properties $\overrightarrow{{{\varvec{x}}}^{\boldsymbol{*}}}$ accurately represent the implant’s mechanical behavior. This optimization guarantees that the simulated deformed shapes align closely with the 3D scan results across all implant sizes considered (*i* = 1 ~ 4).

Afterward, we proceeded to evaluate the optimized material properties $\overrightarrow{{{\varvec{x}}}^{\boldsymbol{*}}}$. To assess these properties, implants were fixed to a vertical surface, considering different attachment conditions, ranging from attachment on 1 to 3 regions, to attachment over the entire region. The deformation of the implants under the influence of gravitational loading was then evaluated by comparing the results of 3D scans with those obtained from FEA.

This integrated optimization, conducted through iterative process, along with validation approach, ensured that the derived material properties $\overrightarrow{{{\varvec{x}}}^{\boldsymbol{*}}}$ accurately represent the implant’s mechanical behavior under both passive deformation and gravity-induced loading. The overall process of characterizing the material properties—combining 3D scanning, finite element simulation, and shape-based optimization—is summarized in Fig 2.

### Model of the Implant’s Anatomical Environment

**Fig 3:**
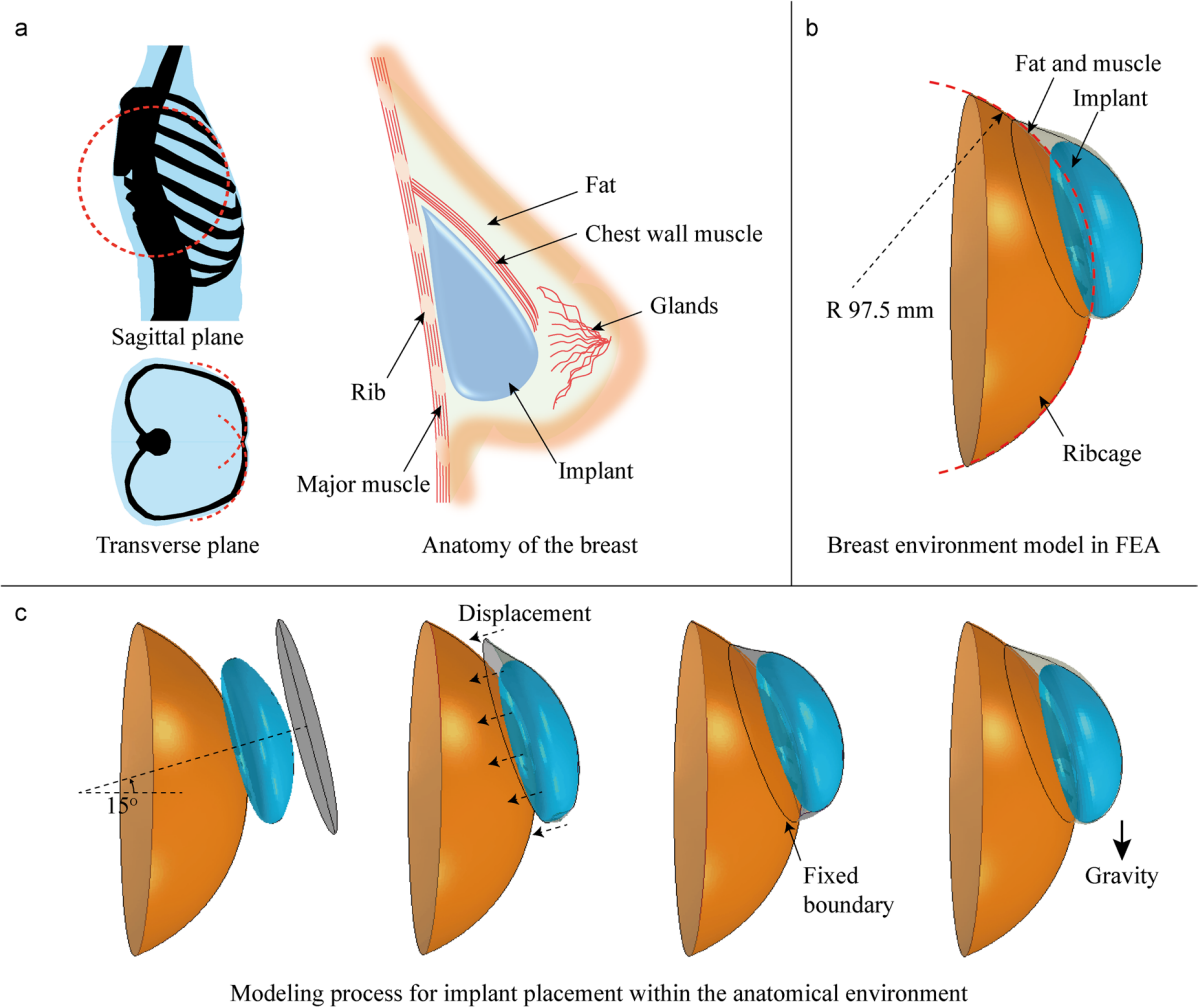
Anatomical context and modeling of a breast implant. **a** Cross-sectional schematic of the ribcage and surrounding soft tissue, highlighting the anatomical region of implant placement. The implant is positioned against the ribcage, showing its spatial relationship with the chest wall and overlying tissue layers. The ribcage beneath the implant is simplified as a spherical shape. **b** The surrounding breast environment is simplified and modeled for Finite Element Analysis. **c** Step-by-step modeling process for implant placement within the anatomical environment. The implant is initially aligned at a 15° angle, then displaced toward the ribcage to mimic surgical positioning. Fixed boundary conditions are applied, and gravity is introduced to allow the implant to settle naturally into the surrounding tissue

The implant is placed beneath the patient’s breast tissues like skin, fat and muscle and is surrounded by the ribcage. Since the body is symmetrical when viewed from the top, the implant is modeled on only one side to simplify the simulation as shown in Fig 3. The portion of the ribcage that contacts the implant has a curved shape, which was simplified for modeling purposes. Based on measurements from MRI images reported in other studies [32–34], the radius of curvature of the contact area on the ribcage was estimated to be approximately 97.5 mm. Consequently, the ribcage was modeled as a hemispherical rigid shell with a radius of 97.5 mm. It is important to note that this approximation does not represent the exact size of the ribcage but was chosen to achieve both simplicity and general applicability. By selecting this value, the simulation maintains relevance across different patient anatomies while ensuring computational efficiency. The ribcage structure was assumed to be significantly stiffer than the implant, with minimal deformation under loading conditions.

During the modeling of implant placement, the implant and the surrounding fat and muscle structure were aligned along a shared axis with an initial tilt from the ground plane. The implant itself was not fixed to the ribcage; instead, the surrounding soft tissue structure was fixed by constraining the displacement of its boundary. Once the boundary of the fat and muscle shell was fixed, gravitational force was applied to the system, allowing the implant to settle into its final position within the anatomical space as shown in Fig 3c.

Through a series of investigations into initial tilt angles, an angle of approximately 15° from the ground plane was found to best reflect the realistic anatomical orientation of the implant in the standing position. This angle was selected to more accurately replicate the natural resting position of the implant within the chest wall, as supported by clinical observations and medical imaging data.

After defining the structural configuration and positioning, the surrounding tissues—skin, fat, and muscle—were modeled using appropriate material properties to accurately simulate the overall mechanical behavior of the breast system. In reality, these properties vary widely across studies and are influenced by factors such as patient age, anatomical location, and skin condition [35]. Additionally, breast tissue has been reported to be significantly softer and more stretchable than silicone implants, exhibiting rubber-like mechanical characteristics [20].

To reduce the complexity of 3D modeling, a simplified representation of the soft tissues was adopted. Our approach is conceptually aligned with the method proposed by Roose et al., who validated soft tissue simulation techniques for breast augmentation using an elastic plate model (30 × 250 × 250 mm) with homogeneous low elastic modulus values ranging from 0.17 to 500 kPa [36]. These values were shown to reasonably reflect the compliant mechanical behavior of breast soft tissues.

In our model, the skin, fat, and muscle were combined into a single homogeneous structure, referred to as the “fat and muscle model,” as illustrated in Fig 3b. The volume of this structure was neglected, and instead, an equivalent thin shell representation—similar to Roose’s plate model—was applied to provide soft tissue coverage while maintaining computational efficiency.

During mechanical properties calibration, we found that assigning excessively stiff mechanical properties led to over-compression of the implant due to strain energy accumulation in the shell. Conversely, overly compliant properties resulted in unrealistic spacing between the implant and ribcage, inconsistent with clinical observations. To address this, we tested a range of mechanical properties and selected an optimal set that preserved the structural integrity of the implant under compression, provided adequate coverage, and minimized the gap between the ribcage, implant, and surrounding soft tissue shell.

As the explained in Material properties section, the material properties of shell were optimized and defined as the material property vector $\overrightarrow{{{\varvec{x}}}^{\boldsymbol{*}}}$. The resulting property **C**_**10**_ of neo-Hookean model was then used in subsequent simulations under compressive and walking loading conditions with different implant sizes. However, the volume fraction of the gel component used in material property vector was no longer applied, as its mechanical behavior of the gel was already captured by the calibrated material model. For simulations in an anatomical environment, the gel volume fraction was assumed to be 1.0, representing an ideally and fully filled implant. The finalized mechanical properties used in the anatomical environment are summarized in Table 1.

**Table 1.** Material model and coefficients of three structures used for FEA in the anatomical environment

Structure	Material model	Material coefficients
Shell	Hyper-elastic neo-Hookean	C_10_ = 100.702 kPa
Gel		C_10_ = 0.025 kPa
Fat and muscle	Linear elastic	E = 208 kPa, *ν* = 0.47

### Loading Condition 1: Compressing

The first loading simulation was designed to model the environment in which the implant undergoes compression loading. This simulation consisted two distinct stages to accurately represent the interactions between the implant, fat and muscle model, and ribcage model.

Stage 1: The primary objective was to position the fat and muscle model so that it would effectively cover and secure the implant on the ribcage model. This was achieved by implementing a specific displacement condition on the boundary nodes of the fat and muscle model.

**Fig 4:**
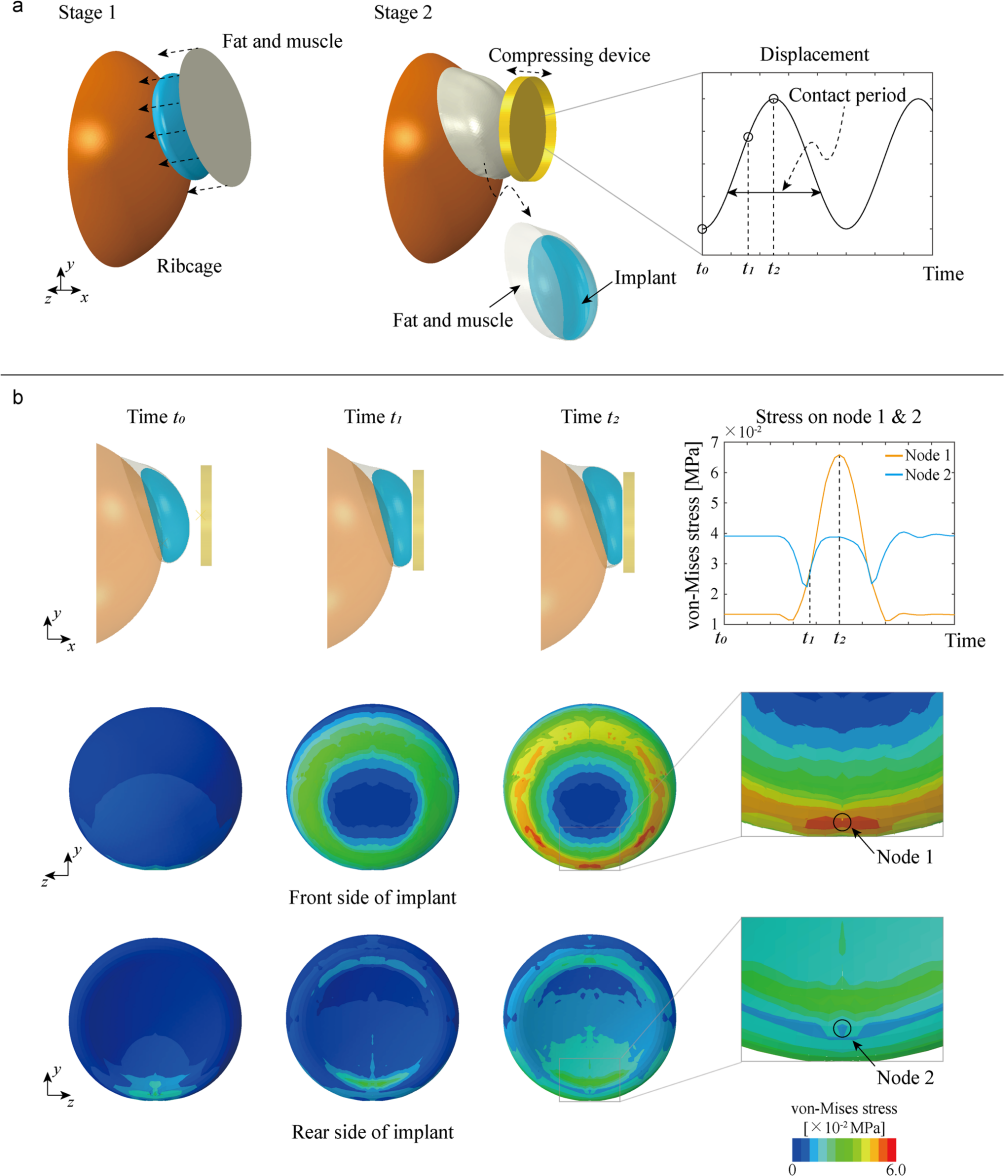
Computational modeling and stress analysis of breast implants under compressing loading condition. **a** Schematic representation of the compressive loading process. Stage 1: The fat and muscle structure is attached to the ribcage, surrounding the implant. Stage 2: A sinusoidal displacement is applied to the compression device, subjecting the implant to periodic compression. **b** Simulation results showing deformation patterns of the implant under varying loads. As the compression device moves under the applied displacement, the implant alternates between contacts and separation from the device over time. During the contact period, high stress concentrations are observed in a circular pattern on the implant surface, correlating with clinically observed rupture location

The fat and muscle model was represented as a circular plate, with its area determined based on the size of the implant. For instance, if the covering area was measured as *A* mm^2^, the area of the circular shell was set to match this value. This resulted in a final radius of $\sqrt{A/\pi }$ mm, ensuring adequate coverage of the implant, as shown in Fig 4a.

Stage 2: After attaching the implant to the ribcage model in Stage 1, a rigid compression device was applied externally to simulate compression conditions. The attached implant from Stage 1 was used as the initial state for Stage 2, ensuring that the stress-free state of the clinically implanted condition was established before applying compression loading. Without setting the stress-free state as the initial state for Stage 2, the residual stress in the fat and muscle model could affect the implant differently from the realistic condition during compression loading.

The compression device was represented as an open-top cylindrical shell with a radius of 45 mm and a height of 10 mm. Its bottom was positioned 10 mm away from the covered implant. This shape and location of the device were carefully determined to adequately cover the deformed implant at the maximum compression depth.

The compression loading condition was applied by setting gravity in negative y direction and by setting the displacement of the compression device in a sinusoidal form, with an amplitude of 10 mm, to compress the implant covered by the fat and muscle model to a maximum depth of 10 mm. During the cycle of applied displacement, the compression device alternately contacts and detaches from the covered implant. We define the time *t*_*0*_ as the moment when the compression device is at its farthest point from the covered implant, *t*_*1*_ as the midpoint during contact between the covered implant and the compression device, and *t*_*2*_ as the deepest point of contact, as shown in Fig 4b.

### Loading Condition 2: Walking

The second loading simulation was designed to replicate the walking environment of a patient with breast implants. To achieve this, datasets from other studies on gait mechanisms were utilized, as they provide valuable insights into motion patterns [37–41]. The dataset with time-tracked position data from body markers, including measurements for three walking speeds, was essential for constructing realistic walking loading conditions in this study. Specifically, position data measured at the xiphoid process—the point closest to the implant, as identified in their study—were used to define the walking environment as a loading condition.

The positional data in the forward walking (x-) direction were considered with uniform velocities of 240, 549, and 986 mm/s. In contrast, the vertical (y-) positional data exhibited periodic motions, with amplitudes of 6.39, 11.07, and 17.31 mm. For the sake of brevity, the horizontal (z-) positional data were not used, as the range of motion in this direction is relatively smaller than the other two, and our model focuses on one side of the patient’s breast.

These velocity and amplitude values are not identical but show consistent trends with previously reported displacement data measured at the suprasternal notch during walking, as described by Haake and Scurr [42]. Vertical movement amplitudes ranging from 18 to 28 mm were observed at walking speeds of approximately 1111.1 mm/s (4 km/h) and 1944.4 mm/s (6 km/h). The comparable relationship between walking speed and vertical displacement in both datasets supports the physiological relevance of the displacement profiles applied in this study.

**Fig 5:**
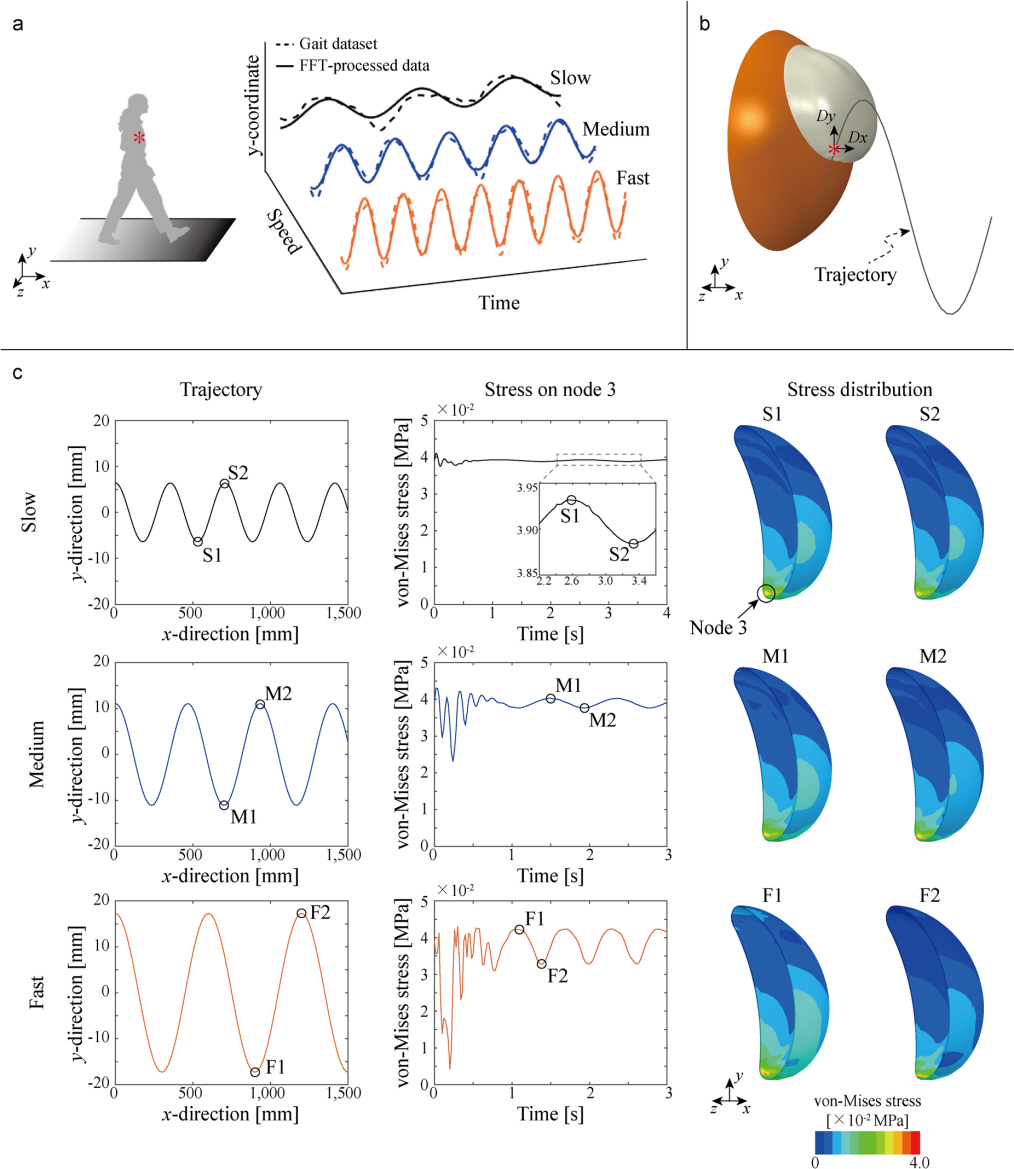
Computational modeling and stress analysis of breast implants under walking loading condition. **a** Representation of the gait cycle, illustrating slow, medium, and fast walking speeds and their corresponding displacement datasets [39]. The Fast Fourier Transform (FFT) is applied to extract periodic components of the gait data. **b** Schematic of the implant subjected to dynamic displacements *Dx* and *Dy* in x- and y- directions respectively. **c** (left) Time-domain displacement profiles showing the applied sinusoidal motions for different walking speeds. (right) Stress response results for implants under walking loading conditions at slow, medium, and fast walking speeds. Higher walking speeds result in greater stress fluctuations, with regions of elevated stress concentration observed on the implant surface, as indicated by the color maps. Node 3 is identified as the point experiencing the highest stress after stabilization, revealing that the lowest location experiences the highest stress levels, and vice versa

For a simplified analysis, the vertical (y-) positional data were processed using a Fast Fourier Transform (FFT) to approximate the positional data based on the main frequency. After determining the main frequency of the vertical position, the amplitude was calculated through a least squares minimization process. The gait dataset and FFT-processed data for walking conditions in vertical (y-) direction at three speeds are illustrated in Fig 5a, and the detail walking conditions are outlined in Table 2.

**Table 2:** Loading conditions as three different speed obtained through FFT process

Direction	Type	Parameter	Speed		
			Slow	Medium	Fast
Forward (x-) direction	Uniform velocity	Velocity [mm/s]	240	549	986
Vertical (y-) direction	Sinusoidal displacement	Amplitude [mm]	6.385	11.070	17.313
		Period [s]	1.474	0.851	0.609

The walking condition was applied by setting gravity in negative y direction and configuring the displacement of the ribcage model in finite element analysis model, denoted as *Dx(t)* and *Dy(t)*, respectively, as shown in Fig 5b. The displacements of ribcage at three different speeds were determined based on the walking conditions outlined in Table 2. The forward (x-) displacement is given by *Dx(t) = vt*, where the *v* is the velocity in the forward (x-) direction and *t* is time. The vertical (y-) displacement is determined given by $Dy\left(t\right)=Acos\left(\frac{2\pi }{T}t\right)$, where *A* is the amplitude and *T* is period, as also specified in Table 2.

### Finite Element Analysis Model

A commercial software package ABAQUS (Dassault Systèmes, 2021 version) was employed for FEA. To capture large deformations under various loading conditions, a dynamic-implicit scheme is used for the analysis.

The implant consists of two distinct parts: the shell and the gel. Both the shell and gel are modeled in 2-dimensional and 3-dimensional forms depending on the applied loading conditions. During the optimization process for determining material properties, only gravity is applied as the loading condition. In this scenario, a full 3-dimensional model is not required; instead, an axis-symmetric 2-dimensional model is sufficient. For the sake of brevity, the 2-dimensional axis-symmetric element (CAX8H) in ABAQUS is used for the implant.

For the processes of vertical hanging and loading conditions 1 and 2, a half-symmetric 3-dimensional model is used. The 3-dimensional tetrahedral element with 10 nodes (C3D10H) in ABAQUS is used for the implant. Approximately 3,000 elements and 12,000 nodes are used in the 2-dimensional analysis, while 110,000 elements and 100,000 nodes are used in the 3-dimensional analysis.

## Results

### Characterization of Material Properties

Following the optimization process to characterize the material properties, the resulting material property vector was applied to the implant model. Specifically, **C**_**10**_, the coefficient of the neo-Hookean material model for the shell, was initially estimated at 91.547 kPa based on experimental data. After optimization process, this coefficient was refined to 100.702 kPa. Additionally, the gel volume fractions of the implant were determined as 0.930, 0.821, 0.837, and 0.972 for implant sizes of 200, 250, 400, and 560 cc, respectively.

The optimized material vector $\overrightarrow{{{\varvec{x}}}^{\boldsymbol{*}}}$, obtained from the optimization process, was evaluated under a hanging condition on a 90-degree wall, as depicted in Fig 2. The observed rippling patterns closely matched the scanned results, verifying the accuracy of the optimization across the various attachment regions. The attachment regions are highlighted with yellow dashed circles in Fig 2.

For the implant attached at one region, three rippling lines were observed splitting from the attached region into the three branches. For the implant attached at two regions, two rippling lines extended from the attached points to the center of the implant surface, with a triangular indented region noted in the upper central area between the two attachment points. This indented region formed because the gel shifted downward due to gravity, making the gel in upper central region less dense and causing the weight of the gel to concentrate on the attachment points. For the implant attached at three regions, three rippling lines were observed extending from the attached points in the direction of gravity. For the implant attached across the entire region, the rippling lines extended from the outer edges to the central region.

### Compressing Condition

The result of stage 1 shows that the implant is placed and secured between the ribcage and the fat and muscle models. As the nodes on the edge of the fat and muscle model move to their corresponding locations of ribcage model, the implant is compressed in the normal direction, as depicted by the black arrow in Fig 4a. After gravity is applied in the downward (negative y-) direction, the implant takes on its final shape within the patient’s body.

In stage 2, the implant is compressed up to 10 mm by movement of the compression device. The von Mises stress distribution on the surface of the implant indicates that the stresses are concentrated on the edge of the contact region between the implant and the compression device. The stress distribution shows higher stress levels as the device moves deeper into the implant.

For a detailed analysis, representative nodes are designated as Node 1 on the front side of the implant and Node 2 on the rear side of the implant, as depicted in Fig 4b. These nodes are selected the maximum stress occurs at the time period. From time *t*_*0*_ to *t*_*1*_, the stress on Node 2 is higher than that on Node 1. However, from the time *t*_*1*_ to *t*_*2*_ the stress on Node 1 becomes higher than that on Node 2. This shift occurs because, as the compression device moves deeper, the compression effect becomes greater than the gravitational effect. As the compression device moves forward and backward repeatedly, this shift in stress occurs repeatedly as well.

### Walking Condition

The results of second loading condition, simulating walking, show how the stress distribution changes over time. Under the three different speed conditions—slow, medium, and fast—the trajectories implied on the ribcage model vary accordingly. The implant secured between the fat and muscle model and ribcage model moves by the movement of ribcage model.

Similar to the compressing condition, a representative node is designated and denoted as Node 3 on the bottom side of the implant, as illustrated in Fig 5c. The stress on Node 3 fluctuates based on the implant’s position. During the transient period (up to approximately 1s), the stress varies, but once this period passes, the stress changes periodically in sync with the implant's position. For instance, when the implant is at position S1 during slow speed, the stress on Node 3 is high; when the implant moves to position S2, the stress on Node 3 is low. This pattern of stress variation is consistent across all three speeds.

However, unlike the compressing condition, the deformed shape of the implant remains largely unchanged because the primary loading is due to gravity and the dynamic moment of the implant’s mass inertia as it is secured on the ribcage model. The amplitude of stress fluctuation at Node 3 is significantly less than that observed under the compressing condition. This small amplitude of stress fluctuation does not occur in just one specific area; rather, the stress distribution across the implant shows minimal change overall.

## Discussion

In this study, we developed and proposed a computational framework to simulate and investigate stresses on breast implants under dynamic loading conditions within the patient’s body. This framework includes determining the implant’s material properties and simulating loading conditions by modeling the geometry to represent the human anatomy.

The material properties of the implants were adjusted and characterized through an optimization process. The gel material was modeled to behave like a liquid, with significantly lower stiffness compared to the shell material. While this low stiffness cannot fully capture the gel’s liquid-like behavior, the FEA results still demonstrated a liquid-like response without employing more complex methods, such as Fluid-Structure Interaction (FSI), which couples Computational Fluid Dynamics (CFD) and FEA [43]. The simulation results for the hanging condition, using these optimized material properties, showed excellent agreement between the scanned deformed shape and the FEA results, as illustrated in Fig 2.

In the validation, vertical rippling lines formed on the surface of the shell due to gravitational forces. When the implant had an even number of attachment points, a horizontal rippling line also developed, reducing the central tension in the vertical direction. The interaction between these vertical and horizontal lines created a complex pattern, resulting in a triangular-shaped indentation on the surface of the shell.

When attached across the entire region, the scanned implant showed a central rippling line, whereas the FEA implant did not. This discrepancy is due to differences in the rippling modes on the surface of the shell. Despite these differences, both the scan and FEA results exhibit symmetry along the implant’s central plane, resulting in overall similar patterns. Although this mode can be modified by adjusting the mesh size in the FEA, this was beyond the scope of our current focus.

To simulate the clinical environment surrounding the implant, the anatomical structures were simplified and modeled. The human ribcage was represented as a hemispherical rigid shell with surface curvature, while the fat and muscle covering the implant were modeled as an elastic thin shell. While this simulation model may not account for the full variability of individual patient anatomies, it provides a practical starting point and can be adapted to analyze other loading scenarios.

Two loading conditions were considered: compression as a specific event and walking as a typical daily event as illustrated as Figs. 4, and 5. Under the compression loading condition, the FEA results showed the von Mises stress distribution as the compression device moved forward over time, from *t*_*0*_ to *t*_*2*_. The highest stress, exceeding 0.06 MPa, was observed at Node 1 along the circular region on the front side of the implant, marked by orange and yellow colored stress ring in Fig 4b.

This observation is significant because the region of highest stress closely corresponds to the area of implant fracture, as shown in Fig 1b. The correlation between the high stress region in the FEA results and the actual fracture location suggests that the simulation accurately predicts potential failure points in the implant under compressive loading. Although the highest stress does not exceed the shell material’s yield stress, repeated exposure to such stress could lead to fatigue fracture over time.

In the second loading condition, walking, the FEA results showed that the highest von Mises stress fluctuates with each step of the patient, based on FFT-processed gait data and the trajectory displacement condition applied to the ribcage model. Since the primary loading differed from that of the compressive condition, the location of the highest stress also shifted, as illustrated in Fig 5. After the initial transient period, the fluctuating stress stabilized at around 0.04 MPa, which is approximately two-thirds of the highest stress level observed under compressive loading condition.

There are several limitations to our proposed procedure for analyzing stress in implant shell. The first limitation is the simplified material model for gel, which did not account for viscosity. While the neo-Hookean elastic model efficiently captured the implant’s response under various loading conditions, the absence of the gel’s viscosity limits its ability to fully represent the gel’s behavior. The second limitation relates to the modeling of the human body. Although ribcage size and the material properties of fat and muscle vary between patients, obtaining precise measurements for each individual is not feasible. Therefore, one single size and elastic model were used to simplify the analysis. The final limitation is the use of simplified loading conditions. External and typical daily loading conditions were chosen for stress analysis, but these conditions vary between patients depending on their lifestyle habits.

For a more accurate analysis that addresses these limitations, a patient’s exact body profile and lifestyle factors should be observed and measured prior to simulation, allowing for customized body modeling and loading conditions based on these individual characteristics. Despite these limitations, our proposed procedure and simulation model effectively predict regions of highest stress under various loading conditions and identify the most vulnerable areas in the implant shell subject to repeated stress. Since the primary cause of implant shell fracture is fatigue due to the repeated stress below the yield stress of shell material, our model can be used to refine implant’s design, reinforcing these vulnerable areas and improving durability.

In summary, we developed a computational framework to predict stress distribution on the implant shell within the patient’s body using FEA under clinically relevant loading conditions. A key feature of this work is the integration of a shape-based optimization procedure to indirectly characterize the nonlinear material properties of the implant, including components like the gel, which are difficult evaluate through direct mechanical test. This inverse optimization process leverages 3D scan data of real implant deformations to calibrate the model and ensure that the mechanical behavior of the simulation reflects actual implant performance. The resulting material properties were validated and then applied in simulations of both compressive and walking-related dynamic loading scenarios.

By identifying areas of high stress and fatigue-prone regions, the proposed framework provides actionable insights for improving implant durability and informing design strategies. While full geometric optimization was beyond the scope of this study, the framework lays essential groundwork for future investigations focused on enhancing implant safety through design refinement. Future work could extend this approach by incorporating more advanced gel material modeling (e.g., viscoelasticity), as well as patient-specific body geometries and lifestyle factors, ultimately enabling more personalized and predictive implant assessments.

## References

[1] Derby, B. M., and M. A. Codner. Textured silicone breast implant use in primary augmentation: core data update and review. *Plast. Reconstr. Surg.* 135(1):113–124, 2015. 10.1097/prs.0000000000000832. 25539301 10.1097/PRS.0000000000000832

[2] Coombs, D. M., et al. Breast augmentation surgery: clinical considerations. *Clevel. Clin. J. Med.* 86(2):111–122, 2019. 10.3949/ccjm.86a.18017. 10.3949/ccjm.86a.18017 30742581

[3] Maxwell, G. P., and A. Gabriel. Breast implant design. *Gland Surg.* 6(2):148, 2017. 10.21037/gs.2016.11.09. 28497018 10.21037/gs.2016.11.09 PMC5409902

[4] Spear, S. L., and M. R. Jespersen. Breast implants: saline or silicone? *Aesthet. Surg. J.* 30(4):557–570, 2010. 10.1177/1090820X10380401. 20829254 10.1177/1090820X10380401

[5] Hillard, C., et al. Silicone breast implant rupture: a review. *Gland Surg.* 6(2):163, 2017. 10.21037/gs.2016.09.12. 28497020 10.21037/gs.2016.09.12 PMC5409893

[6] Wu, H.-H., et al. Rupture of 40-year-old silicone gel breast implants: a case report. *BMC Geriat.* 23(1):589, 2023. 10.1186/s12877-023-04293-3. 10.1186/s12877-023-04293-3 PMC10517471 37742002

[7] Sigurdson, L., and D. H. Lalonde. MOC-PS (SM) CME Article: breast reconstruction. *Plast. Reconstr. Surg.* 121(1S):1–12, 2008. 10.1097/01.prs.0000294668.32874.18. 18182962 10.1097/01.prs.0000294668.32874.18

[8] McCarthy, C. M., et al. Patient satisfaction with postmastectomy breast reconstruction: a comparison of saline and silicone implants. *Cancer*. 116(24):5584–5591, 2010. 10.1002/cncr.25552. 21136577 10.1002/cncr.25552

[9] Salzman, M. J. Silent rupture of silicone gel breast implants: high-resolution ultrasound scans and surveys of 584 women. *Plast. Reconstr. Surg.* 149(1):7–14, 2022. 10.1097/prs.0000000000008632. 34936597 10.1097/PRS.0000000000008632 PMC8687613

[10] Juanpere, S., et al. Imaging of breast implants—a pictorial review. *Insights Into Imaging*. 2(6):653–670, 2011. 10.1007/s13244-011-0122-3. 22347984 10.1007/s13244-011-0122-3 PMC3259319

[11] Hölmich, L. R., et al. Incidence of silicone breast implant rupture. *Arch. Surg.* 138(7):801–806, 2003. 10.1001/archsurg.138.7.801. 12860765 10.1001/archsurg.138.7.801

[12] Nava, M. B., et al. How to prevent complications in breast augmentation. *Gland Surg.* 6(2):210, 2017. 10.21037/gs.2017.04.02. 28497025 10.21037/gs.2017.04.02 PMC5409896

[13] Rancati, A. O., et al. Silicone implant rupture. In: Aesthetic breast augmentation revision surgery: from problem to solution, edited by R. de Vita, et al., . Cham: Springer, 2022, pp. 101–110. 10.1007/978-3-030-86793-5.

[14] Marotta, J. S., et al. Silicone gel breast implant failure: evaluation of properties of shells and gels for explanted prostheses and meta-analysis of literature rupture data. *Ann. Plast. Surg.* 49(3):227–247, 2002. 12351970 10.1097/00000637-200209000-00001

[15] Necchi, S., et al. Failure of silicone gel breast implants: is the mechanical weakening due to shell swelling a significant cause of prostheses rupture? *J. Mech. Behav. Biomed. Mater.* 4(8):2002–2008, 2011. 10.1016/j.jmbbm.2011.06.019. 22098899 10.1016/j.jmbbm.2011.06.019

[16] Swarts, E., et al. Rupture of poly implant prothèse silicone breast implants: an implant retrieval study. *Plast. Reconstr. Surg.* 131(4):480e–489e, 2013. 10.1097/prs.0b013e3182818a00. 23249979 10.1097/PRS.0b013e3182818a00

[17] Fraldi, M., et al. Stealthy role of size-driven stresses in biomechanics of breast implants capsular contracture. *J. Mech. Behav. Biomed. Mater.* 64:199–208, 2016. 10.1016/j.jmbbm.2016.07.028. 27508316 10.1016/j.jmbbm.2016.07.028

[18] Santecchia, E., et al. A review on fatigue life prediction methods for metals. *Adv. Mater. Sci. Eng.* 2016(1):9573524, 2016. 10.1155/2016/9573524.

[19] Lee, H. S., et al. Two-step sub-modeling framework for thermomechanical fatigue analysis of solder joints in DRAM module. *Microelectron. Reliab.*160:115469, 2024. 10.1016/j.microrel.2024.115469.

[20] Myung, Y., et al. Finite element analysis of long-term changes of the breast after augmentation mammoplasty: implications for implant design. *Arch. Plast. Surg.* 46(04):386–389, 2019. 10.5999/aps.2019.00346. 31336428 10.5999/aps.2019.00346 PMC6657194

[21] Kovar, M., et al. Validation of breast implant finite element model. *Comput. Methods Biomech. Biomed. Eng.* 20(sup1):S109–S110, 2017. 10.1080/10255842.2017.1382885. 10.1080/10255842.2017.1382885 29088587

[22] Do, Y., and D.-N. Kim. Investigating the effect of slit patterns on the deformation of thin-walled tubes under side impact. *J. Mech. Sci. Technol.* 36(11):5649–5655, 2022. 10.1007/s12206-022-1027-4.

[23] Cho, H., and D.-N. Kim. Controlling the stiffness of bistable kirigami surfaces via spatially varying hinges. *Mater. Des.*231:112053, 2023. 10.1016/j.matdes.2023.112053.

[24] Yun, G., J. Lee, and D.-N. Kim. Stability of mixed overlapping elements in incompressible analysis. *Comput. Methods Appl. Mech. Eng.*412:116104, 2023. 10.1016/j.cma.2023.116104.

[25] Kang, D., J. M. Hur, and D.-N. Kim. Mechanical meta-sheets with independently tunable Poisson’s ratio and coefficient of thermal expansion. *Mater. Des.*244:113187, 2024. 10.1016/j.matdes.2024.113187.

[26] Ye, L., et al. The effect of lens shape, zonular insertion and finite element model on simulated shape change of the eye lens. *Ann. Biomed. Eng.* 2024. 10.1007/s10439-024-03491-3. 38503945 10.1007/s10439-024-03491-3 PMC11247046

[27] Guo, Z., and L. Sluys. Application of a new constitutive model for the description of rubber-like materials under monotonic loading. *Int. J. Solids. Struct.* 43(9):2799–2819, 2006. 10.1016/j.ijsolstr.2005.06.026.

[28] Bae, J.-H., and S.-H. Chang. A study on the mechanical behavior of silicone-organically modified montmorillonite composite under human body simulated environment. *Compos. Sci. Technol.* 85:90–97, 2013. 10.1016/j.compscitech.2013.06.008.

[29] Noor, S.N.A.M. and J. Mahmud. *Modelling and computation of silicone rubber deformation adapting neo-hookean constitutive equation*. in *2015 Fifth International Conference on Communication Systems and Network Technologies*. 2015. IEEE. 10.1109/csnt.2015.276.

[30] Jebur, Q., et al. Hyperelastic models for the description and simulation of rubber subjected to large tensile loading. *Arch. Mater. Sci. Eng.* 2021. 10.5604/01.3001.0015.0256.

[31] Melly, S. K., et al. A review on material models for isotropic hyperelasticity. *Int. J. Mech. Syst. Dyn.* 1(1):71–88, 2021. 10.1002/msd2.12013.

[32] Song, Y., et al. Predictive parameters for selection of electronic tissue compensation radiotherapy in early-stage breast cancer patients after breast-conserving surgery. *Oncotarget*. 7(22):32835, 2016. 10.18632/oncotarget.9054. 27147569 10.18632/oncotarget.9054 PMC5078055

[33] Alghufaili, A. H., L. Shanmugarajah, and L. K. Kumaraswamy. Correlating the depth of compensation to the 3-D shape of the breast to achieve homogeneous dose distribution using the electronic tissue compensation treatment technique. *Med. Dosim.* 44(1):30–34, 2019. 10.1016/j.meddos.2018.01.001. 29525491 10.1016/j.meddos.2018.01.001

[34] Chen, S. N., P. Ramachandran, and P. Deb. Dosimetric comparative study of 3DCRT, IMRT, VMAT, Ecomp, and Hybrid techniques for breast radiation therapy. *Radiat. Oncol. J.* 38(4):270, 2020. 10.3857/roj.2020.00619. 33389982 10.3857/roj.2020.00619 PMC7785843

[35] Eder, M., et al. Comparison of different material models to simulate 3-D breast deformations using finite element analysis. *Ann. Biomed. Eng.* 42:843–857, 2014. 10.1007/s10439-013-0962-8. 24346816 10.1007/s10439-013-0962-8

[36] Roose, L., et al. Validation of different soft tissue simulation methods for breast augmentation. In: International congress series,Amsterdam: Elsevier, 2005. 10.1016/j.ics.2005.03.126.

[37] Wang, L., et al. Silhouette analysis-based gait recognition for human identification. *IEEE Trans. Pattern Anal. Mach. Intell.* 25(12):1505–1518, 2003. 10.1109/tpami.2003.1251144.

[38] Moore, J. K., S. K. Hnat, and A. J. van den Bogert. An elaborate data set on human gait and the effect of mechanical perturbations. *PeerJ*.3:e918, 2015. 10.7717/peerj.918. 25945311 10.7717/peerj.918 PMC4419525

[39] Schreiber, C., and F. Moissenet. A multimodal dataset of human gait at different walking speeds established on injury-free adult participants. *Sci. Data*. 6(1):111, 2019. 10.1038/s41597-019-0124-4. 31270327 10.1038/s41597-019-0124-4 PMC6610108

[40] Topham, L. K., et al. Human body pose estimation for gait identification: A comprehensive survey of datasets and models. *ACM Comput. Surv.* 55(6):1–42, 2022. 10.1145/3533384.

[41] Van Criekinge, T., et al. A full-body motion capture gait dataset of 138 able-bodied adults across the life span and 50 stroke survivors. *Sci. Data*. 10(1):852, 2023. 10.1038/s41597-023-02767-y. 38040770 10.1038/s41597-023-02767-y PMC10692332

[42] Haake, S., and J. Scurr. A method to estimate strain in the breast during exercise. *Sports engineering*. 14:49–56, 2011. 10.1007/s12283-011-0071-6.

[43] Bungartz, H.-J., and M. Schäfer. Fluid-structure interaction: modelling, simulation, optimisation. Berlin: Springer Science & Business Media, 2006.

